# High serum levels of N-epsilon-carboxymethyllysine are associated with poor coronary collateralization in type 2 diabetic patients with chronic total occlusion of coronary artery

**DOI:** 10.1186/s12872-022-02694-7

**Published:** 2022-06-22

**Authors:** Le-Ying Li, Shuai Chen, Fei-Fei Li, Zhi-Ming Wu, Ying Shen, Feng-Hua Ding, Xiao-Qun Wang, Wei-Feng Shen, Qiu-Jing Chen, Yang Dai, Lin Lu

**Affiliations:** 1 Department of Cardiovascular Medicine, Ruijin Hospital, Shanghai Jiao Tong University School of Medicine, 197 Rui Jin Road II, Shanghai, 200025 People’s Republic of China; 2 Institute of Cardiovascular Diseases, Shanghai Jiao Tong University School of Medicine, Shanghai, People’s Republic of China

**Keywords:** Type 2 diabetes mellitus, Chronic total occlusion, Coronary collateral vessel, N-epsilon-carboxymethyllysine

## Abstract

**Background:**

The formation of advanced glycation end-products (AGEs) is a crucial risk factor for the pathogenesis of cardiovascular diseases in diabetes. We investigated whether N-epsilon-carboxymethyllysine (CML), a major form of AGEs in vivo, was associated with poor coronary collateral vessel (CCV) formation in patients with type 2 diabetes mellitus (T2DM) and chronic total occlusion (CTO) of coronary artery.

**Methods:**

This study consisted of 242 T2DM patients with coronary angiographically documented CTO. Blood samples were obtained and demographic/clinical characteristics were documented. The coronary collateralization of these patients was defined according to Rentrop or Werner classification. Serum CML levels were evaluated using ELISA assay. Receiver operating characteristic curve and multivariable regression analysis were performed.

**Results:**

242 patients were categorized into poor CCV group or good CCV group (107 vs. 135 by the Rentrop classification or 193 vs. 49 by the Werner classification, respectively). Serum CML levels were significantly higher in poor CCV group than in good CCV group (110.0 ± 83.35 vs. 62.95 ± 58.83 ng/ml by the Rentrop classification and 94.75 ± 78.29 ng/ml vs. 40.37 ± 28.69 ng/ml by Werner classification, both P < 0.001). Moreover, these CML levels were also significantly different across the Rentrop and Werner classification subgroups (P < 0.001). In multivariable logistic regression, CML levels (P < 0.001) remained independent determinants of poor CCV according to the Rentrop or Werner classification after adjustment of traditional risk factors.

**Conclusions:**

This study suggests that higher serum CML level is associated with poor collateralization in T2DM patients with CTO.

## Introduction

Diabetes causes impairment of coronary collateral vessel (CCV) formation in response to occlusion of a patent artery in patients with coronary artery disease [1]. Good CCV formation is functionally important to provide myocardial protection against infarction and increase patients’ survival rates [2]. Previous studies have evidenced that dysregulation of pro-angiogenic and anti-angiogenic elements contributes to poor CCV in ischemic tissues in diabetes [3, 4]. Pathophysiologically, this pathologic feature is caused by increased formation and accumulation of advanced glycation end products (AGEs) and augmentation of oxidative stress and inflammatory reactions [3, 4].

In the diabetic milieu, AGEs play a central role in the pathophysiology of vascular complications including post-ischemia angiogenesis and arteriogenesis impairment [5–7]. Engagement of the receptor for AGEs (RAGE) with AGEs activates pathways in endothelial cells or macrophages, leading to augmented oxidative stress and inflammation in ischemic myocardial tissues, ending up with poor collateralization [5, 6].

N-epsilon-carboxymethyllysine (CML) is the most abundant AGEs in vivo [8]. In diabetic condition, CML-modified proteins may exhibit structural alterations, thereby resulting in dysfunction of these proteins. Moreover, CML-modified protein also activates RAGE pathway, jointly accelerating the development of various vasculopathies (i.e., macrovascular and microvascular diseases) in diabetes [5, 9–13]. However, the relation of CML to coronary collateralization in diabetic patients with chronic total occlusion (CTO) remains unclear.

In the present study, we performed coronary angiography and used the Rentrop and the Werner classification to assess the condition of CCV formation in T2DM patients with CTO. The serum levels of CML were evaluated via ELISA in the participants. Our study was purposed to explore the relationship between serum CML levels and coronary collateralization in T2DM patients with CTO.

## Methods

### Study population and grouping

The study protocol was approved by the Ruijin Hospital and Shanghai Jiao Tong University School of Medicine Ethics Committee, and written informed consent was obtained from all participants.

**Fig. 1:**
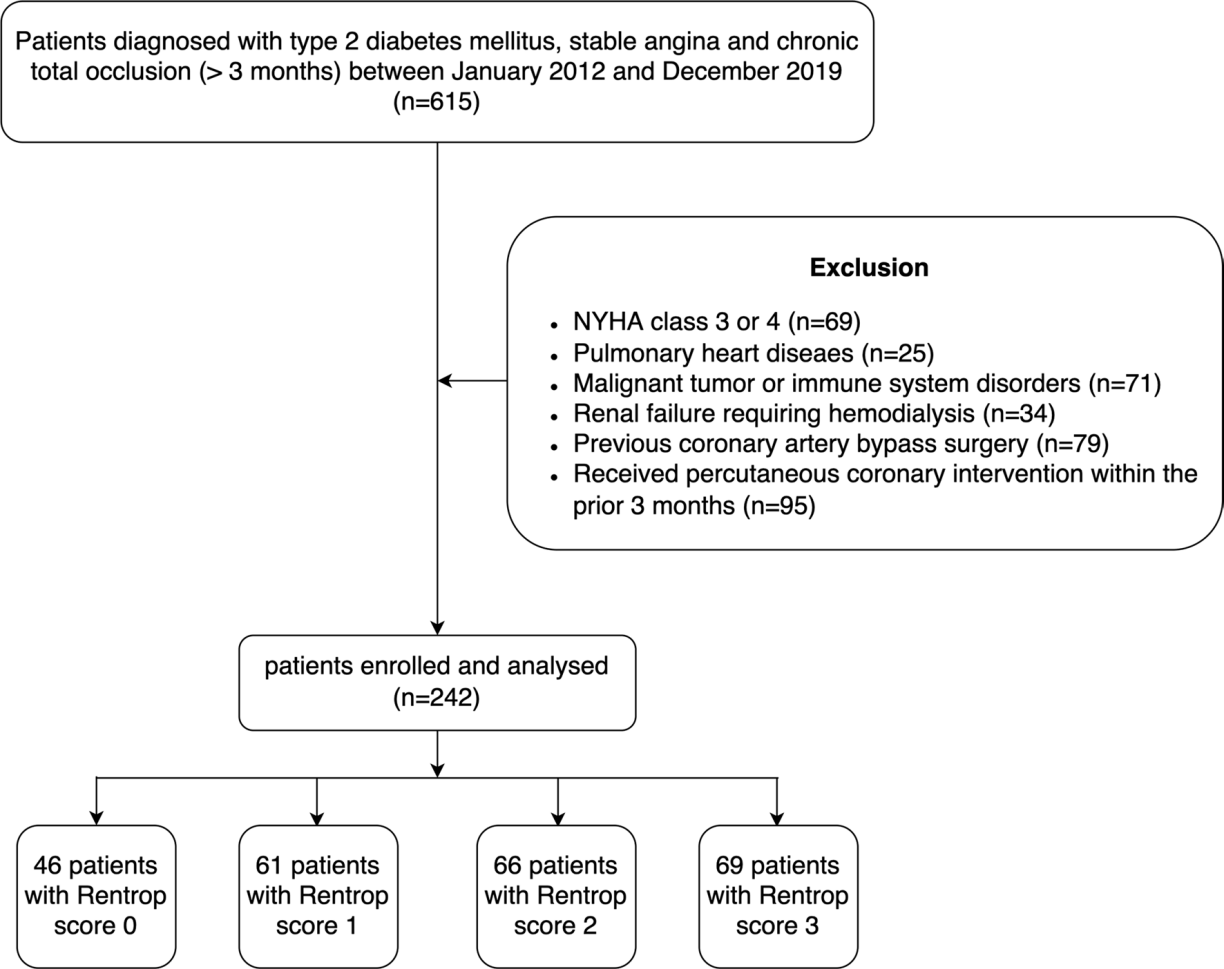
Flowchart of patient enrollment

A total of 615 T2DM patients with stable angina and at least one lesion with coronary angiographic total occlusion were enrolled between January 2012 and December 2019. This inclusion criterion was based on long-standing knowledge that a severe coronary artery obstruction was a prerequisite for spontaneous collateral recruitment [14]. Stable angina was diagnosed according to the criteria recommended by the American College of Cardiology/American Heart Association [15]. For the purpose of this research, we excluded patients with chronic heart failure (n = 69), pulmonary heart disease (n = 25), malignant tumors or immune system disorders (n = 71), renal failure requiring hemodialysis (n = 34) as well as patients who had a history of coronary artery bypass grafting (n = 79) or received percutaneous coronary intervention within the prior 3 months (n = 95). The remaining 242 diabetic patients with stable angina and CTO (> 3 months) were eligible and categorized in this study (Fig. 1). The diagnosis of T2DM and hyperlipidemia were made according to the 2016 guideline of ESC [16] and 2017 update of ESC/EAS on PCSK 9 inhibition [17]. Type 1 diabetes was excluded by measurement of C-peptide levels. Detailed information regarding demographics, clinical manifestation and medications used was obtained.

### Coronary angiography

Coronary angiography was performed through the femoral or radial approach. All angiograms were reviewed by two experienced interventional cardiologists, according to lesion classification scheme of the American College of Cardiology/American Heart Association [18]. Both of them were blinded to the study protocol and clinical data. Any differences in interpretation were judged by a third reviewer.

The condition of CCV was determined using the Rentrop classification as in previous studies [19–21], as follows: grade 0 = no collaterals, grade 1 = side branch filling of the recipient artery without visualization of the epicardial artery, grade 2 = partial filling of the main epicardial coronary artery, grade 3 = complete filling of the main epicardial coronary artery [22]. The Werner classification was graded as: coronary collateral (CC) 0, no visible connection between the donor and the recipient coronary artery; CC1, thread-like connection between the donor and the recipient coronary artery; CC2, side-branch like connection between the donor and the recipient coronary artery [23].

Patients with Rentrop 0–1 or CC 0–1 were categorized as poor CCV group and those with Rentrop 2–3 or CC 2 were referred to good CCV group. Thus, the present study contained 242 patients with 107 in poor CCV group according to the Rentrop classification and 193 according to the Werner classification.

### Sample acquisition and biochemical measurement

Blood samples were obtained from patients undergoing angiography after 12 h of fasting. Samples were collected by centrifugation at the speed of 3000 rpm for 10 min. All serum samples were stored at − 80 °C until analysis. Serum glucose, glycosylated hemoglobin A1c (HbA1c), blood urea nitrogen, creatinine, uric acid, and lipid profiles were measured with standard laboratory techniques on a Hitachi 912 Analyzer (Roche Diagnostics, Germany). Modified estimated glomerular filtration rate (eGFR) was calculated.

### CML Quantification

Serum CML levels were measured with Cell BioLabs CML Competitive ELISA kit (STA-816) according to the manufacturer’s instructions. The CML ELISA kit used a colorimetric immunoassay method and CML levels of samples were determined by comparing samples OD values with a standard curve of gradient dilution of CML-modified BSA, in which higher CML modification correlates with lower OD signal. The final CML levels were shown with ng/ml unit by calculation of CML-modified BSA/CML. The inter-assay variation was controlled in an acceptable range.

### Statistical analysis

Continuous variables are presented as mean ± standard deviation (SD), and categorical data are summarized as frequency (percentage). For categorical clinical variables, differences between groups were evaluated by the chi-square test followed by Bonferroni’s correction. For continuous variables, normal distribution was evaluated with the Kolmogorov–Smirnov test. Differences among groups were analyzed by one-way analysis of variance (ANOVA) followed by *post-hoc* analysis (Bonferroni’s correction). Receiver operating characteristic (ROC) curves were plotted to assess the power of CML for detecting poor CCV and to compare its power when CML was added or not added into combined risk factors (Model 2 and Model 4, versus Model 1 and Model 3). Area under the curve (AUC) was compared using the DeLong method. Risk factors for CAD including gender, age, body mass index (BMI), hypertension, smoking, HbA1c, eGFR and high-sensitivity C reactive protein (hsCRP) were recruited into multivariable logistic regression analyses with or without CML measurements to assess determinants for poor CCV. All analyses used 2-sided tests with alpha value set at 0.05. All statistical analyses were performed with IBM SPSS Version 26 for Mac (IBM SPSS Inc, Chicago, IL, USA) and Prism 9 for macOS (1994–2021 GraphPad Software, LLC).

## Results

### Baseline characteristics

The characteristics and parameters of patients with poor CCV or good CCV categorized according to the Rentrop or the Werner classification are presented in Table 1. Patients of poor CCV group according to the Rentrop classification were older and more smokers, had lower ratio of male and hypertension, with poor glycemic control, exhibited higher serum levels of creatinine and hsCRP but lower eGFR values in comparison with those of good CCV group (for all comparison, P < 0.05). Whereas, more Smoking, poor glycemic control, high levels of LDL-C, apoB, and creatinine, and low eGFR were manifested in poor CCV group according to the Werner classification (for all comparison, P < 0.05).

**Table 1:** (A) Characteristics and parameters of patients categorized by the Rentrop classification; (B) characteristics and parameters of patients categorized by the Werner classification

(A)			
	Poor CCV (n = 107)	Good CCV (n = 135)	P value
Male, n (%)	74 (69.16)	114 (84.44)	**0.005**
Age, years	67.31 ± 11.22	64.19 ± 10.10	**0.024**
BMI, kg/m^2^	25.25 ± 3.74	24.98 ± 3.34	0.546
Smoking, n (%)	41 (38.32)	34 (25.19)	**0.036**
Hypertension, n (%)	71 (66.36)	106 (78.52)	**0.041**
SBP, mmHg	134.93 ± 21.58	136.06 ± 19.96	0.672
DBP, mmHg	73.45 ± 10.55	75.11 ± 11.43	0.246
FBG, mmol/L	8.44 ± 3.41	7.69 ± 2.77	0.060
HbA1c, %	6.95 ± 1.43	6.37 ± 1.58	**0.003**
Dyslipidemia, n (%)	29 (27.10)	22 (16.30)	0.056
Triglyceride, mmol/L	1.77 ± 0.93	1.70 ± 1.22	0.650
Total cholesterol, mmol/L	3.98 ± 1.29	3.87 ± 1.08	0.496
LDL-C, mmol/L	2.33 ± 1.05	2.25 ± 0.89	0.516
HDL-C, mmol/L	1.01 ± 0.20	1.06 ± 0.28	0.133
ApoA, g/L	1.12 ± 0.22	1.15 ± 0.23	0.312
ApoB, g/L	0.80 ± 0.27	0.77 ± 0.23	0.367
Lp(a), g/L	0.36 ± 0.86	0.30 ± 0.29	0.411
BUN, mmol/L	7.18 ± 4.84	6.93 ± 3.77	0.648
Serum creatinine, μmol/L	101.74 ± 53.74	85.10 ± 66.51	**0.037**
eGFR, ml·min^−1^·1.73 m^−2^	68.51 ± 20.80	85.17 ± 20.49	** < 0.001**
UA, μmol/L	348.69 ± 104.15	338.62 ± 97.02	0.438
hsCRP, mg/L	14.58 ± 33.21	7.14 ± 20.63	**0.034**
*Medication, n (%)*			
ACE inhibitor/ARB	59 (55.14)	61 (45.19)	0.124
β-blocker	81 (75.70)	90 (66.67)	0.125
Nitrate	45 (42.06)	53 (39.26)	0.660
Calcium channel blocker	20 (18.69)	24 (17.78)	0.855
Statins	80 (74.77)	105 (77.78)	0.583
Antidiabetic therapy	107 (100.00)	135 (100.00)	**/**

(B)			
	Poor CCV (n = 193)	Good CCV (n = 49)	P value
Male, n (%)	145 (75.13)	43 (87.76)	**0.082**
Age, years	65.96 ± 11.03	64.04 ± 9.23	0.263
BMI, kg/m^2^	25.04 ± 3.56	25.35 ± 3.34	0.575
Smoking, n (%)	69 (35.75)	6 (12.24)	**0.002**
Hypertension, n (%)	137 (70.98)	40 (81.63)	0.152
SBP, mmHg	135.80 ± 21.01	134.59 ± 19.38	0.715
DBP, mmHg	74.19 ± 10.90	75.10 ± 11.75	0.608
FBG, mmol/L	8.03 ± 3.20	7.71 ± 2.43	0.509
HbA1c, %	6.76 ± 1.55	6.10 ± 1.37	**0.007**
Dyslipidemia, n (%)	45 (23.32)	6 (12.24)	0.116
Triglyceride, mmol/L	1.74 ± 0.97	1.72 ± 1.52	0.912
Total cholesterol, mmol/L	3.98 ± 1.24	3.68 ± 0.89	0.112
LDL-C, mmol/L	2.35 ± 1.01	2.04 ± 0.69	**0.048**
HDL-C, mmol/L	1.02 ± 0.24	1.09 ± 0.27	0.080
ApoA, g/L	1.13 ± 0.22	1.17 ± 0.23	0.187
ApoB, g/L	0.80 ± 0.26	0.71 ± 0.18	**0.022**
Lp(a), g/L	0.34 ± 0.67	0.28 ± 0.32	0.547
BUN, mmol/L	7.10 ± 4.49	6.52 ± 1.89	0.377
Serum creatinine, μmol/L	96.90 ± 67.79	74.98 ± 17.30	0.026
eGFR, ml·min^−1^·1.73 m^−2^	75.04 ± 22.85	88.68 ± 15.23	** < 0.001**
UA, μmol/L	346.85 ± 102.83	327.98 ± 78.58	0.232
hsCRP, mg/L	11.73 ± 29.00	5.32 ± 17.18	0.140
*Medication, n (%)*			
ACE inhibitor/ARB	99 (51.30)	21 (42.86)	0.338
β-blocker	141 (73.06)	30 (61.22)	0.116
Nitrate	76 (39.38)	22 (44.90)	0.517
Calcium channel blocker	36 (18.65)	8 (16.33)	0.837
Statins	144 (74.61)	41 (83.67)	0.257
Antidiabetic therapy	193 (100.00)	49 (100.00)	/

Data are mean ± SD or number (%); P values were in bold if P < 0.05

CCV coronary collateral vessel, BMI body mass index, SBP systolic blood pressure, DBP diastolic blood pressure, FBG fasting blood glucose, HbA1c glycosylated hemoglobin A1c, LDL-C low-density lipoprotein cholesterol, HDL-C high-density lipoprotein cholesterol, BUN blood urea nitrogen, UA uric acid, eGFR estimated glomerular filtration rate, hsCRP high-sensitivity C reactive protein

### Serum CML levels are significantly increased in patients with poor CCV

**Fig. 2:**
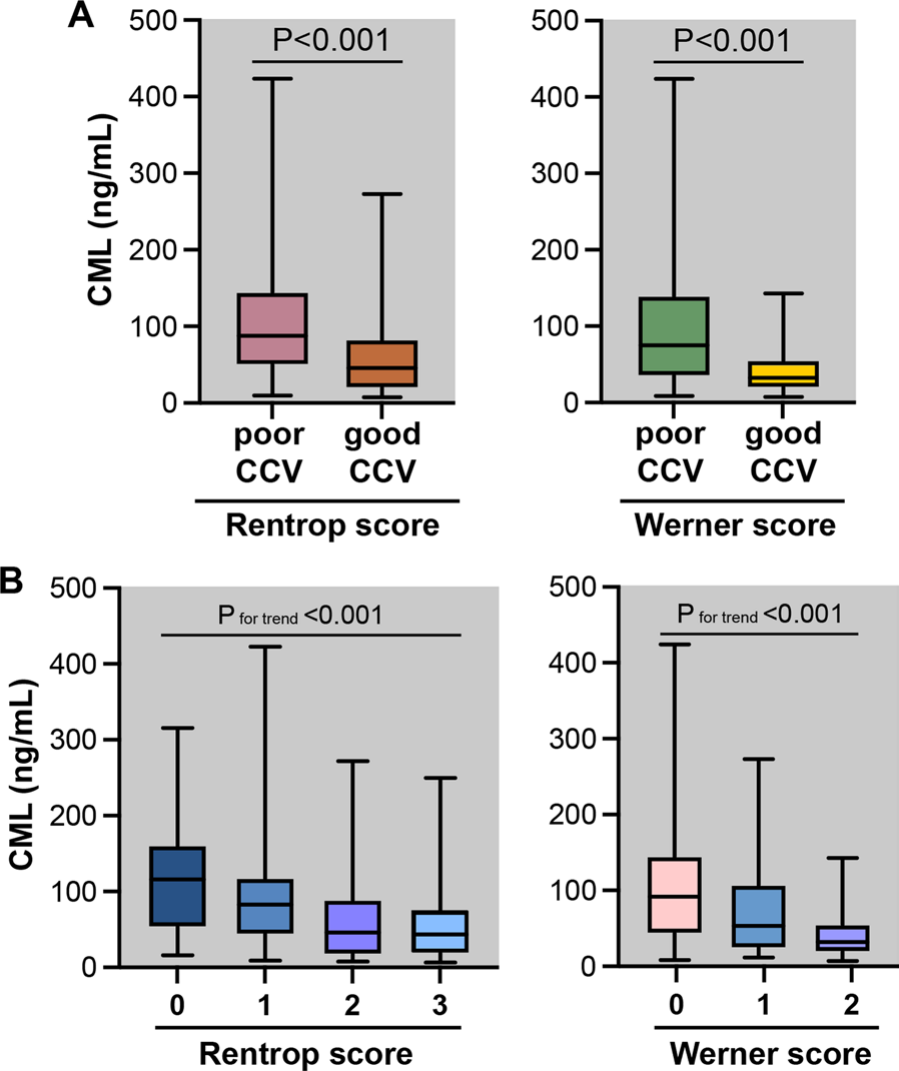
Serum CML levels. **a** CML levels in poor and good CCV groups categorized by the Rentrop score and the Werner score; **b** CML level distributions in the Rentrop scores and the Werner scores

Serum CML levels were significantly increased in poor CCV group (110.0 ± 83.35 ng/ml by the Rentrop classification and 94.75 ± 78.29 ng/ml by the Werner classification) than in good CCV group (62.95 ± 58.83 ng/ml by the Rentrop classification and 40.37 ± 28.69 ng/ml by the Werner classification, both P < 0.001) (Fig. 2a). CML levels were also significantly different across the subgroups categorized according to the Rentrop classification (Rentrop score 0, 120.8 ± 75.12 ng/ml; Rentrop score 1, 101.8 ± 88.78 ng/ml; Rentrop score 2, 67.01 ± 64.78 ng/ml; Rentrop score 3, 59.07 ± 52.70 ng/ml, respectively) and the Werner score (CC0, 107.50 ± 84.43; CC1, 75.69 ± 65.51; CC2, 40.90 ± 29.98, respectively) (both P for trend < 0.001) (Fig. 2b).

The percentage of poor CCV increased stepwise from the lowest tertile to the highest tertile of CML in both classifications before and after adjustment of multiple variables including gender, age, BMI, smoking, hypertension, HbA1c, eGFR and hsCRP levels (all P for trend < 0.001) (Table 2).

**Table 2:** Odds ratio of poor collateralization in diabetic patients

Tertiles of CML (n, range ng/ml)	Poor CCV, n (%)	Crude OR (95% CI)	^a^Adjusted OR (95% CI)
*Patients categorized according to the Rentrop classification*			
Tertile 1 (n = 80, < 38.76)	19 (23.75)	1	1
Tertile 2 (n = 80, 38.76–95.75)	37 (46.25)	2.763 (1.404–5.437) ^*^	2.556 (1.161–5.624) ^*^
Tertile 3 (n = 82, > 95.75)	51 (62.20)	5.282 (2.672–10.441) ^**^	6.802 (2.980–15.526)^**^
Per tertile	–	2.278 (1.626–3.192)^**^	2.610 (1.729–3.941)^**^
P value for tertile trend	< 0.001	< 0.001	< 0.001
*Patients categorized according to the Werner classification*			
Tertile 1 (n = 80, < 38.76)	53 (66.25)	1	1
Tertile 2 (n = 80, 38.76–95.75)	62 (77.50)	1.755 (0.871–3.534)	1.206 (0.529–2.748)
Tertile 3 (n = 82, > 95.75)	78 (95.12)	9.934 (3.285–30.038)^**^	9.701 (2.898–32.472)^**^
Per tertile	/	2.683 (1.719–4.189) ^**^	2.510 (1.534–4.106) ^**^
P value for tertile trend	< 0.001	< 0.001	< 0.001

*CCV* coronary collateral vessel, *CI* confidence interval, *OR* odds ratio

*P < 0.05; **P < 0.001

^a^Multiple-adjustment for gender, age, body mass index, hypertension, smoke, HbA1c, estimated glomerular filtration rate, total-to-HDL cholesterol ratio and serum level of high sensitive C reactive protein

**Fig. 3:**
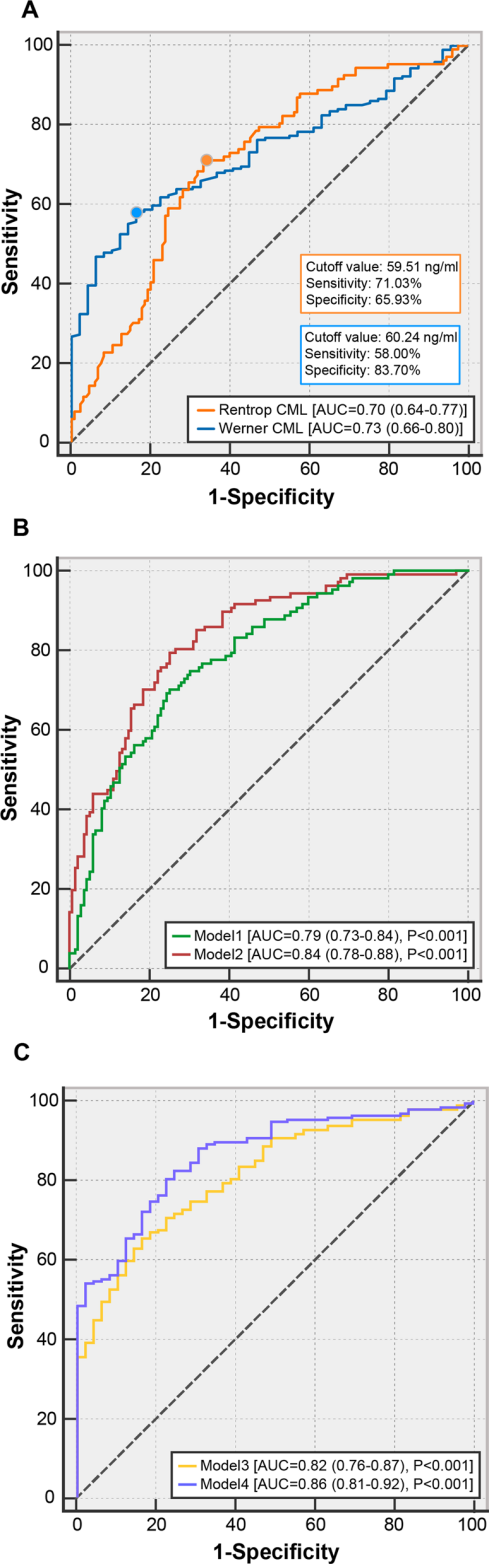
ROC curves for detecting poor collateralization. **a** ROC of CML for determining poor collaterals categorized by the Rentrop score and the Werner score; **b** ROC of Model 1 and Model 2; **c** ROC of Model 3 and Model 4

ROC curve for detecting poor CCV exhibited that AUC was 0.70 (95% CI 0.64–0.77, P < 0.001) for CML by the Rentrop classification and 0.73 (95% CI 0.66–0.80, P < 0.001) by the Werner classification. The cutoff values were 59.51 ng/ml and 60.24 ng/ml according to Youden’s index with a diagnostic sensitivity of 71.03% or 58% and specificity of 65.93% or 83.7%, respectively (Fig. 3a).

### Multivariable analysis

Multivariate logistic regression analysis was performed to ascertain independent determinants of poor CCV. In Model 1, we included major parameters in Table 1, including gender, age, BMI, hypertension, smoking, HbA1c, hypercholesterolemia, eGFR and hsCRP. The results showed that less hypertension, smoking, poor glycemic control, low eGFR and high hsCRP levels in Model 1 (the Rentrop classification), and smoking, poor glycemic control, high Total-to-HDL cholesterol ratio, low eGFR and high hsCRP in Model 3 (the Werner classification) were independent determinants for poor collateralization. After adjustment for these variables, serum CML levels remained independently associated with poor CCV (OR = 1.999, 95% CI 1.530–2.613, P < 0.001 in Model 2 [the Rentrop classification] (Table 3A), and OR = 1.827, 95% CI 1.361–2.453, P < 0.001 in Model 4 [the Werner classification] (Table 3B). The calibrations of all models were good. The addition of CML significantly improved predictive performance with an increase of Nagelkerke R^2^ by 12.7% and 9.2% (both P < 0.001). In addition, ROC curve for models showed that addition of CML effectively elevated the AUC value (Model 2, AUC = 0.84, 95% CI 0.78–0.88 P < 0.001 vs. Model 1, AUC = 0.79, 95% CI 0.73–0.84 P < 0.001) (P = 0.016) (Fig. 3b) (Model 4, AUC = 0.86, 95% CI 0.81–0.92 P < 0.001 vs. Model 3, AUC = 0.82, 95% CI 0.76–0.87 P < 0.001) (P = 0.006) (Fig. 3c).

**Table 3:** (A) Logistic regression analyses to determine risk factors for poor collateralization according to the Rentrop classification in diabetic patients; (B) logistic regression analyses to determine risk factors for poor collateralization according to the Werner classification in diabetic patients

	Variables	OR (95% CI)	P value
*(A)*			
Model 1	Male	0.816 (0.375–1.777)	0.609
Nagelkerke R^2^ = 0.303	Age per 10 years	1.099 (0.816–1.478)	0.535
Hosmer–Lemeshow test:	BMI	1.070 (0.976–1.174)	0.149
P = 0.651	Hypertension	0.422 (0.217–0.821)	**0.011**
	Smoking	2.249 (1.189–4.255)	**0.013**
	HbA1c	1.324 (1.084–1.619)	**0.006**
	Hypercholesterolemia	1.527 (0.603–3.865)	0.372
	Total-to-HDL cholesterol ratio	0.888 (0.696–1.134)	0.343
	eGFR	0.967 (0.951–0.983)	** < 0.001**
	Log hsCRP	1.134 (1.003–1.282)	**0.045**
Model 2	Male	0.742 (0.310–1.775)	0.503
Nagelkerke R^2^ = 0.430	Age per 10 years	1.124 (0.821–1.539)	0.465
Hosmer–Lemeshow test:	BMI	1.058 (0.959–1.167)	0.261
P = 0.981	Hypertension	0.323 (0.154–0.677)	**0.003**
	Smoking	1.910 (0.973–3.750)	0.06
	HbA1c	1.309 (1.057–1.620)	**0.014**
	Hypercholesterolemia	1.276 (0.494–3.296)	0.615
	Total-to-HDL cholesterol ratio	0.864 (0.658–1.135)	0.294
	eGFR	0.964 (0.947–0.981)	** < 0.001**
	Log hsCRP	1.182 (1.032–1.355)	**0.016**
	Log2 CML	1.999 (1.530–2.613)	** < 0.001**
*(B)*			
Model 3	Male	0.874 (0.291–2.628)	0.810
Nagelkerke R^2^ = 0.306	Age per 10 years	0.919 (0.620–1.363)	0.675
Hosmer–Lemeshow test:	BMI	0.977 (0.872–1.095)	0.693
P = 0.911	Hypertension	0.442 (0.181–1.082)	0.074
	Smoking	4.514 (1.707–11.935)	**0.002**
	HbA1c	1.351 (1.027–1.778)	**0.032**
	Hypercholesterolemia	0.851 (0.223–3.241)	0.813
	Total-to-HDL cholesterol ratio	1.489 (1.056–2.098)	**0.023**
	eGFR	0.970 (0.948–0.993)	**0.012**
	Log hsCRP	1.248 (1.047–1.487)	**0.013**
Model 4	Male	0.662 (0.209–2.091)	0.482
Nagelkerke R^2^ = 0.398	Age per 10 years	0.910 (0.599–1.383)	0.659
Hosmer–Lemeshow test:	BMI	0.955 (0.845–1.080)	0.465
P = 0.456	Hypertension	0.379 (0.146–0.984)	**0.046**
	Smoking	3.736 (1.375–10.150)	**0.010**
	HbA1c	1.293 (0.983–1.702)	0.067
	Hypercholesterolemia	0.587 (0.144–2.391)	0.457
	Total-to-HDL cholesterol ratio	1.538 (1.052–2.249)	**0.026**
	eGFR	0.973 (0.950–0.996)	**0.021**
	Log hsCRP	1.290 (1.076–1.547)	**0.006**
	Log2 CML	1.827 (1.361–2.453)	** < 0.001**

Model 1 and 3, adjusted for conventional cardiovascular factors; Model 2 and 4, adjusted for the factors included in Model 1 and 3 with the addition of CML; P values were in bold if P < 0.05

*BMI* body mass index, *HbA1c* glycosylated hemoglobin A1c, *HDL* high-density lipoprotein, *eGFR* estimated glomerular filtration rate, *hsCRP* high sensitive C reactive protein

## Discussion

Patients with diabetes often exhibit poor coronary collateralization after ischemia [1]. Our study has demonstrated that serum CML levels are significantly increased in T2DM CTO patients with poor CCV as compared with those with good CCV. Serum CML levels are inversely correlated with the Rentrop and Werner score in these patients. In logistic regression analysis, serum CML level is an independent determinant of poor CCV in patients with T2DM and CTO. Our study supported the notion that increased CML levels contribute to poor coronary collateralization in T2DM patients with CTO.

Hyperglycemia-associated formation of AGEs and subsequent engagement of AGEs with RAGE causes augmented oxidative stress and robust inflammation, leading to diabetic cardiovascular complications (macrovascular and microvascular vasculopathies), and robust production of AGEs, which in return results in a vicious cycle [3–6].

Among various AGEs, CML modifications predominate in vivo in diabetes [8]. Previous studies have evidenced the impact of CML in the pathogenesis of cardiovascular diseases associated with diabetes. Specific AGEs including CML are associated with incident cardiovascular events with T2DM [24, 25]. Glycation and CML levels in skin collagen predict future 10-year progression of diabetic retinopathy and nephropathy in controls and in intervention and complication patients of type 1 diabetes [26]. Serum levels of AGEs (mainly CML) are associated with impaired renal function and pathogenic mechanisms of chronic kidney disease [27, 28]. Circulating Levels of CML are closely related to central obesity and inflammation [29], carotid diameter [30], and differentiate early to moderate Alzheimer's disease [31]. Moreover, plasma AGEs, in particular CML levels, are found to be related to the severity and prognosis of CHF [32]. Consistent with above-mentioned studies, our study has showed that high CML levels are associated with poor collateralization in type 2 diabetic patients with CTO. Our findings have further added novel information regarding CML, indicating that CML aggravates the pathogenesis coronary vasculopathies and meanwhile impairs the repairing mechanisms of CCV formation in ischemic tissues. It is also worthy of noting that collateral vessels can be found even in nonviable myocardium. Our future study may further investigate the potential correlation between the CML levels and the actual presence of myocardial viability.

In our study, hypertension was an antagonistic factor of poor CCV. It is interesting to mention that a dozen previous studies have probed the relationship between hypertension and CCV formation in myocardial ischemia, with inconsistent results. For instance, a meta-analysis showed that among 18 studies about post-ischemia CCV, 7 studies associated hypertension with better CCV formation, and 9 studies were opposite [33]. We believe that this phenomenon could be explained as that moderate elevation of blood pressure may increase the pressure gradient in coronary collateral vessels, facilitating collateral vessels development.

Sufficient evidence has revealed that reduction of AGEs levels may be effective to alleviate diabetic vasculopathies [34]. Alagebrium, capable of breaking cross-link structure in AGEs, targets the miR-27b/TSP-1 signaling pathway to attenuate CML-induced endothelial dysfunction [35]. Soluble RAGE (sRAGE) is a RAGE isoform generated through alternative splicing or shedding from cell membrane. sRAGE combines with AGEs to prevent the engagement of AGEs with RAGE and subsequent activation of RAGE pathway [34]. Animal studies have shown that administration of sRAGE remarkably stabilizes atherosclerotic plaque, and inhibits inflammatory factors such as cyclooxygenase-2 (COX-2), VCAM-1, and monocyte chemoattractant protein-1 (MCP-1), thereby attenuating atherosclerosis progression [36–38]. Moreover, antioxidants (e.g., vitamin C, vitamin E), ACEIs, ARBs and statins are capable of inhibiting AGEs formation [32]. These data jointly suggest that inhibition of renin-angiotensin system, modulation of dyslipidemia, AGE inhibition, RAGE pathway inhibition and oxidative stress reduction are therapeutic strategies for preventing cardiovascular complications in diabetes, partially through antagonizing AGEs formation. Future studies may investigate whether inhibition of CML affects coronary collateralization after myocardial ischemia.

## Limitations

We recognize limitations in our study. First, this study is a cross-sectional study, these relationships are correlational and not necessarily causal due to the non-randomized nature of the study, and all the enrolled patients were from a single center. Second, the Rentrop and Werner classifications are not most precise ways for evaluation of coronary collateralization. It is more accurate by calculating collateral flow index, which requires measurement of pressure within aorta and the distal culprit segment at the same time. Last, CMLs have been traditionally quantified by gas chromatography/mass spectrometry (GC/MS). What’s more, the risk for confounding bias is present in the process of statistical analysis. Thereby, the correlation between CML and poor collateralization needs further support by prospective studies.

## Conclusion

In conclusion, our results suggest that higher CML is associated with poor collateralization in T2DM patients with CTO.

## Data Availability

The datasets generated and/or analysed during the current study are not publicly available due to patients’ privacy protection, but are available from the corresponding author on reasonable request.
